# Does posterior tibial slope laterality exist? A matched cohort study between ACL-injured and non-injured knees

**DOI:** 10.1186/s40634-023-00702-z

**Published:** 2023-12-07

**Authors:** Keiji Tensho, Daiki Kumaki, Kazushige Yoshida, Hiroki Shimodaira, Hiroshi Horiuchi, Jun Takahashi

**Affiliations:** 1 Department of Orthopedic Surgery, Shinshu University School of Medicine, 3-26-1, Asahi, Matsumoto, Nagano 390-8621 Japan; 2 Department of Rehabilitation, Shinshu University Hospital, 3-26-1, Asahi, Matsumoto, Nagano 390-8621 Japan

**Keywords:** Anterior cruciate ligament, Posterior tibial slope, Laterality

## Abstract

**Purpose:**

The purpose of this study is to examine 1) the degree and frequency of laterality in posterior tibial slope (PTS) with control and anterior cruciate ligament (ACL) injury groups and 2) the laterality of PTS between sides of injury and dominant legs in patients with primary ACL injuries.

**Methods:**

A total of 187 consecutive patients with clinically diagnosed noncontact ACL injuries and an age- and sex-matched 1:1 control group were identified. PTS was measured using three different methods (aPTS = anterior PTS, mPTS = middle PTS, pPTS = posterior PTS) on a lateral knee radiograph. PTS of the left and right sides were compared between the patients in the control and ACL-injured groups, and between the injured and non-injured and dominant and non-dominant legs among the patients in the ACL-injured group. The patients with a difference in PTS of ≥ 3° in mPTS were selected. The percentages were compared between left and right between and among both groups, and between the injured and non-injured, and non-dominant and dominant leg, for the ACL group. Multiple regression analysis was performed to analyze the factors influencing the degree of mPTS.

**Results:**

Both control (Right vs Left: aPTS; 9.0 ± 2.5 vs 10.5 ± 3.0, mPTS; 6.6 ± 2.3 vs 8.1 ± 2.7, pPTS; 4.0 ± 2.4 vs 5.6 ± 2.8, respectively, p < 0.01) and ACL injury groups (Right vs Left: aPTS; 10.6 ± 3.0 vs 12.6 ± 2.9, mPTS; 7.6 ± 2.6 vs 9.5 ± 2.6, pPTS; 5.9 ± 3.0 vs 8.0 ± 3.0, respectively, p < 0.01) had a significantly greater PTS on the left than on the right side, and the ACL group had a significantly greater PTS than the control group on both the left and right sides. In the ACL group, PTS was greater on the injured and the non-dominant leg than on the non-injured and the dominant leg. The percentage of patients with a PTS difference of ≥ 3° was significantly greater on the left, injured, and non-dominant leg (95.3% vs 4.7%, 73.8% vs 26.2%, 86.1% vs 13.9%, respectively, p < 0.01). Only the left leg had a significant influence on PTS in the multivariate analysis.

**Conclusion:**

There was laterality in PTS within control and ACL injury groups, and this information is of benefit for effective treatment of ACL injuries.

**Level of evidence:**

Level III.

## Introduction

Anterior cruciate ligament (ACL) tears are among the most common knee joint ligament injuries. There are many known risk factors for ACL injury, and patient-unique bone morphology has also been shown to play a significant role [3].

Numerous clinical studies have shown that a large posterior tibial slope (PTS) is a major risk factor for ACL injury [21, 22, 27] because it promotes anterior displacement of the tibia during weight bearing [7, 20] and increases the stress on the ACL [4], which also affects rotational instability [2]. Furthermore, there is an increased load on the graft. Hence, numerous reports have suggested that PTS could be a risk factor for graft failure [23] and re-injury [1, 6, 10, 11]. In addition, contralateral injury [9] and several types of meniscal injury [12], such as ramp lesion [17] and medial/lateral root tear [13, 18], have also been reported to be significantly affected by the PTS. Hence, this bony parameter has been investigated in various studies.

**Fig. 1:**
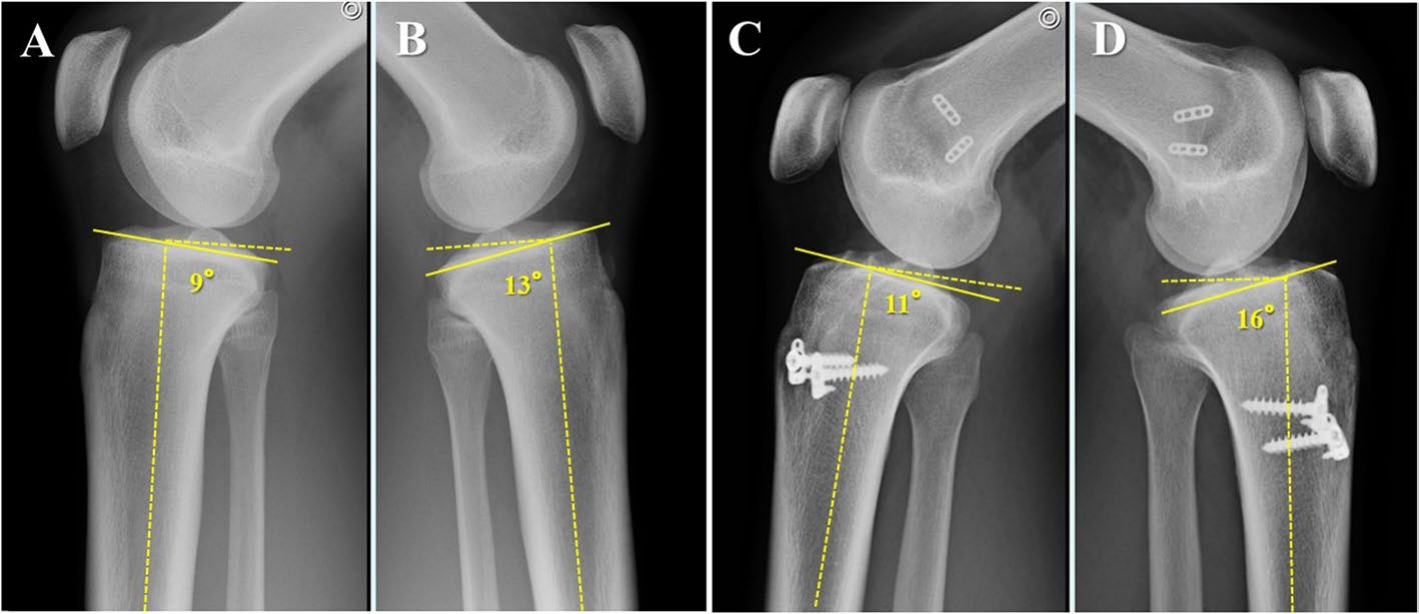
A 17-year-old female had an ACL injury in her left knee. A lateral view of the knee shows a PTS of (**A**) 9° on the right and (**B**) 13° on the left, with a 4°-difference between the right and left. A 23-year-old woman with bilateral ACL injury after ACL reconstruction. A lateral view of the knee shows a PTS of (**C**) 11° on the right and (**D**) 16° on the left with a 5° left–right difference. The right knee joint is clinically stable, whereas the left was re-injured

Although PTS has attracted much attention, there are few reports on the laterality of PTS. In our own experience, we have seen several patients with left–right differences in the degree of PTS (Fig. 1), and these patients showed primary ACL injury or re-injury of the graft on the side with a large PTS. This is relevant because if laterality exists in the same patient, it can be used to evaluate the effect of PTS on ACL injury and significantly impact how to prevent postoperative re-injury. The purpose of this study was to examine 1) the degree and frequency of left–right differences in PTS between patients with primary ACL injuries and the control group and 2) the degree and frequency of differences in PTS based on the side of injury and the dominant leg using simple lateral radiographs of both knees in patients with primary ACL injuries. We hypothesized that there exists a left–right difference in PTS, and PTS on the injured side would be greater compared to the un-injured side.

## Materials and methods

### Patient selection and study design

This retrospective cross-sectional study was conducted in accordance with the principles of the Declaration of Helsinki. It was approved by the Shinshu University Ethics Committee (February 22, 2023 / No. 5764). Informed consent was obtained by providing details of the study on the hospital website and allowing those who did not wish to participate in the study to refuse (opt-out approach). Patients who underwent primary ACL reconstruction at our institution between July 2012 and December 2019 were included in this study.

The exclusion criteria were as follows: (1) contact injury, (2) contralateral ACL injury, (3) multi-ligament injury, (4) skeletally immature, (5) knee with osteoarthritis, (6) previous knee surgery, (7) no preoperative true lateral knee-joint X-ray images for both sides, and (8) missing patient demographic information including height, weight, gender, age, and dominant leg. The dominant leg was defined as the foot that kicks a ball [25]. Additionally, a control group of age- and sex-matched patients were selected from individuals who had undergone initial diagnostic lateral knee-joint X-ray imaging for both sides between 2011 and 2022 at our hospital. All patients were skeletally mature and excluded those with previous surgery, ligamentous injuries, meniscus injuries, osteoarthritis, fractures, or other structural problems that were diagnosed on subsequent evaluations.

### Radiographic measurements

**Fig. 2:**
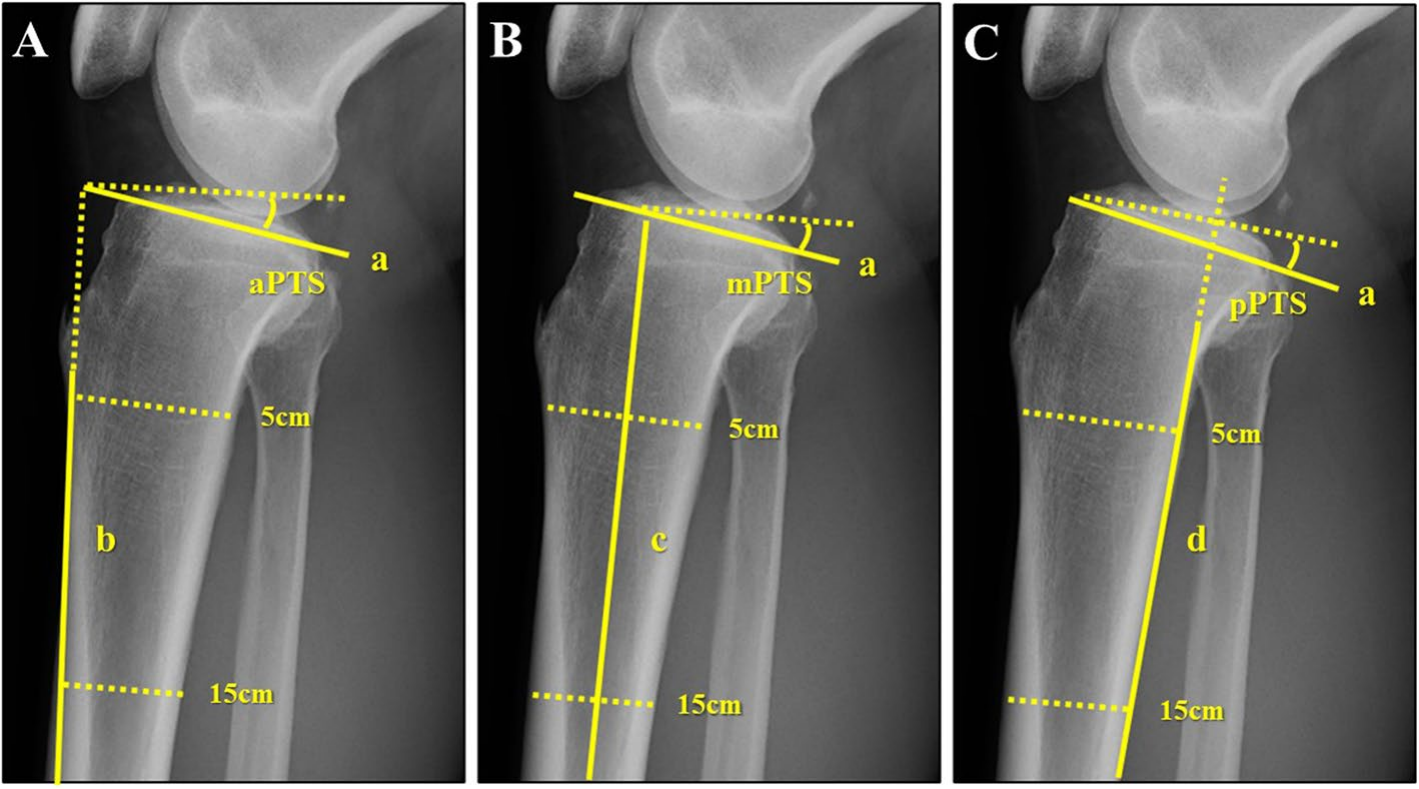
The three methods used for the measurement of PTS as the cosine angle formed by the tangent to the medial tibial plateau (Line a) and perpendicular to each reference axis. **A** aPTS: the angle between lines a and b. **B** mPTS: the angle between line a and c. **C** pPTS: the angle between line a and d. a = tangent line to the medial tibial plateau, b = line connecting two points 5 cm and 15 cm distal to the knee joint on the anterior cortical line of the proximal tibia, c = line connecting two points 5 cm and 15 cm distal to the knee joint on the midpoint of the anterior/posterior cortical surface of the proximal tibia, d = line connecting two points 5 cm and 15 cm distal to the knee joint on the posterior cortical line of the proximal tibia. PTS = posterior tibial slope, aPTS = anterior PTS, mPTS = middle PTS, pPTS = posterior PTS

This study only used a plain true lateral view of the knee joint to evaluate PTS. To minimize the influence of different measurement methods in PTS evaluation, the following three methods were used [24]: PTS was defined as the angle between the tangent line to the medial tibial plateau and a line perpendicular to the three anatomic reference axes (the anterior cortical axis [ACA], proximal anatomic axis [PAA], and posterior cortical axis [PCA]). It was denoted as anterior PTS, middle PTS, and posterior PTS (aPTS, mPTS, pPTS, respectively) (Fig. 2). ACA was defined as a line connecting two points, 5 cm and 15 cm distal to the knee joint, on the anterior cortical line of the proximal tibia; PAA was defined as a line connecting the midpoint of the anteroposterior cortical diameter, which was 5 cm and 15 cm distal to the knee joint. PCA was defined as a line connecting two points, 5 cm and 15 cm distal to the knee joint, on the posterior cortical line of the tibia. Measurements were performed by a single orthopedic surgeon (KT) who was blinded to patient characteristics (right or left difference, leg dominance, with or without ACL injury, etc.). The left–right differences in PTS were compared between the control and ACL injury groups. Similar comparisons were made between the injured and non-injured leg and the dominant and nondominant leg in the ACL injury group. As there are numerous studies showing a difference of 2–3 degrees in PTS with and without ACL injury [21, 22, 27], patients with a difference in PTS of ≥ 3° were considered to have a meaningful PTS laterality. These patients were classified into subgroups with or without laterality. The proportion of large left–right PTS difference was compared between the control and ACL-injured groups, as well as between the injured and non-injured side and dominant and nondominant leg in the ACL injury group.

### Statistical analysis

A priori sample size analysis with a two-tailed Student’s t test determined that 176 knees were needed to detect a moderate effect size (d = 0.30) with a power of 0.80 and an alpha of 0.05. G*Power 3 software (Heinrich Heine University, Dusseldorf, Germany) was used for power analyses. The PTS (aPTS, mPTS, and pPTS) for twenty patients were measured twice by two observers (KT and DK) with > 2 weeks interval between each measurement. The inter- and intraobserver reliability intraclass correlation coefficient (ICC) for aPTS, mPTS, and pPTS are listed in Table 1. Data are presented as means ± SD. After data normality was established, independent t-tests were used to compare the PTS within and between groups in the control and ACL groups, as well as between the injured and non-injured leg and the dominant and non-dominant leg within the ACL group. χ^2^ tests were used to compare categorical variables. A univariate linear analysis was performed with mPTS as the dependent variable and age, sex, height, weight, injured side, dominant leg, and left leg as independent variables. Next, a multiple linear regression analysis was performed for those with a *p*-value of ≤ 0.2 in the single regression analysis. All statistical analyses were performed using the freeware EZR software version 1.38 (Saitama Medical Center, Jichi Medical University, Saitama, Japan), and a *p*-value < 0.05 was considered statistically significant.

**Table 1:** Inter-and intraobserver reliabilities for aPTS, mPTS, and pPTS

Measurement	aPTS (95%CI)	mPTS (95%CI)	pPTS (95%CI)
Interobserver	0.92 (0.81–0.94)	0.86 (0.79–0.89)	0.91 (0.61–0.97)
Intraobserver	0.89 (0.81–0.93)	0.87 (0.73–0.92)	0.87 (0.71–0.96)

*PTS* posterior tibial slope, *aPTS* anterior PTS, *mPTS* middle PTS, *pPTS* posterior PTS

## Results

**Fig. 3:**
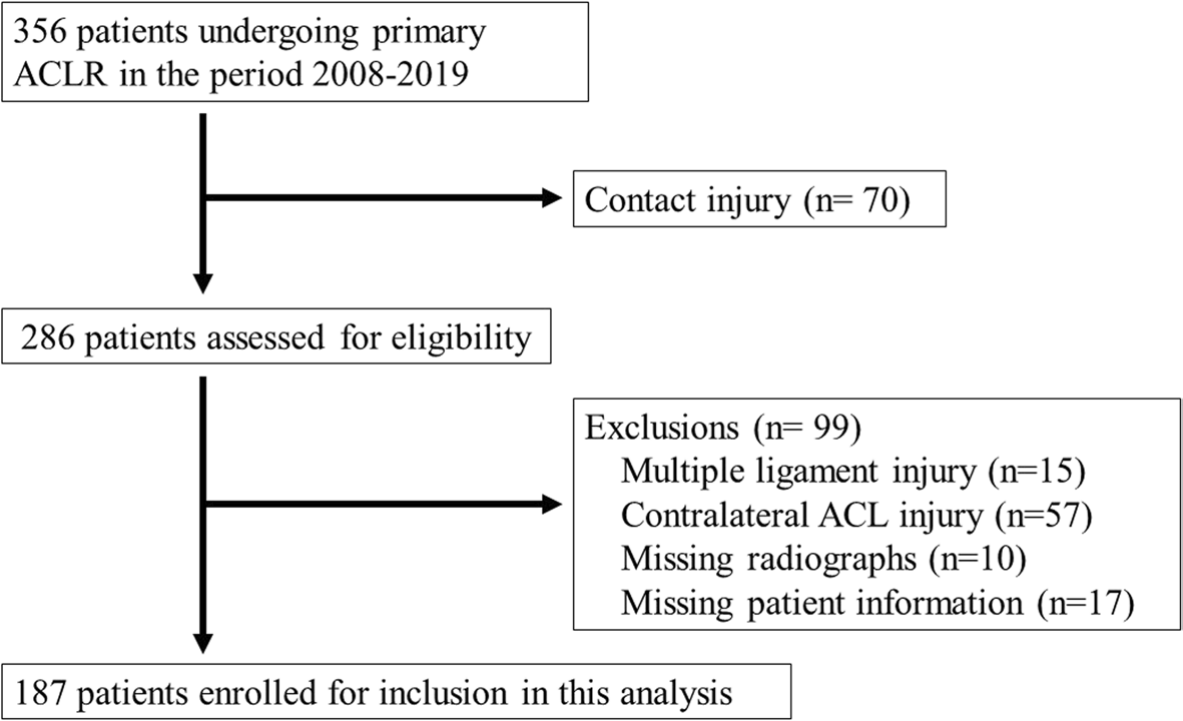
Flow chart outlining the patient selection process. ACLR = anterior cruciate ligament reconstruction

Figure 3 shows a patient flow chart for the ACL groups. Table 2 shows the patients’ background characteristics. Each patient group comprised 187 cases.

**Table 2:** Patient demographics^a^

	Control group (*n* = 187)	ACL injury group (*n* = 187)
Age, years (range)	30.1 ± 14.3 (13–60)	30.0 ± 14.3 (14–55)
Male/female, n	62/125	62/125
Height, cm (range)	N/A	162.9 ± 7.8 (148–178)
Weight, kg (range)	N/A	60.2 ± 10.2 (43–80)
Injury side	N/A	Right 71 (37.9%)
		Left 116 (62.1%)
Leg Dominance	N/A	Right 169 (90.3%)
		Left 18 (9.7%)

*ACL* anterior cruciate ligament

^a^Data are expressed as mean ± SD (range)

All measurements are shown in Tables 3, 4, and 5. The aPTS, mPTS, and pPTS were significantly larger on the left than on the right side in both the control (Right vs Left: aPTS; 9.0 ± 2.5 vs 10.5 ± 3.0, mPTS; 6.6 ± 2.3 vs 8.1 ± 2.7, pPTS; 4.0 ± 2.4 vs 5.6 ± 2.8, respectively, *p* < 0.01 between all measurements) and ACL (Right vs Left: aPTS; 10.6 ± 3.0 vs 12.6 ± 2.9, mPTS; 7.6 ± 2.6 vs 9.5 ± 2.6, pPTS; 5.9 ± 3.0 vs 8.0 ± 3.0, respectively, *p* < 0.01 between all measurements) groups, and all PTSs were significantly larger in the ACL group than in the control group on both sides (*p* < 0.01 between all measurements). Side to side differences were not significantly observed between the groups (Control vs ACL: aPTS; 1.5 ± 2.3 vs 2.0 ± 2.7, *p* = 0.06, mPTS; 1.5 ± 2.4 vs 1.9 ± 2.5, *p* = 0.07, pPTS; 1.5 ± 2.4 vs 2.0 ± 2.7, *p* = 0.05). In the ACL injury group, the PTS was significantly greater on the injured than on the non-injured leg (Injury vs Non-Injury: aPTS; 12.0 ± 3.2 vs 11.1 ± 3.0, mPTS; 9.0 ± 2.9 vs 8.0 ± 2.9, pPTS; 7.5 ± 3.2 vs 6.4 ± 3.2, *p* < 0.01 between all measurements) and the non-dominant than on the dominant leg (Dominant vs Non-Dominant: aPTS; 12.0 ± 3.2 vs 11.1 ± 3.0, mPTS; 9.0 ± 2.9 vs 8.0 ± 2.9, pPTS; 7.5 ± 3.2 vs 6.4 ± 3.2, *p* < 0.01 between all measurements). In comparing males and females, aPTS/pPTS on the left side and aPTS/pPTS on the non-injured side showed differences between males and females. However, there were no significant differences in any other parameters.

**Table 3:** PTS comparison between control and ACL injury groups^a^

	Control	*p*-value of R vs L	ACL injury	*p*-value of R vs L	*p*-value of C vs A
aPTS					
Right	9.0 ± 2.5 (2.5–14.1)	** < .01**	10.6 ± 3.0 (7.8–13.4)	** < .01**	** < .01**
Left	10.5 ± 3.0 (2.4–19.3)		12.6 ± 2.9 (7.8–13.4)		** < .01**
Side to side Difference	1.5 ± 2.3 (-3.5–7.3)		2.0 ± 2.7 (-4.9–10.3)		NS
mPTS					
Right	6.6 ± 2.3 (1.5–12.2)	** < .01**	7.6 ± 2.6 (7.8–13.4)	** < .01**	** < .01**
Left	8.1 ± 2.7 (0.2–16)		9.5 ± 2.6 (1.9–18)		** < .01**
Side to side Difference	1.5 ± 2.4 (-4.3–9.8)		1.9 ± 2.5 (-4.5–9.7)		NS
pPTS					
Right	4.0 ± 2.4 (-2–10.4)	** < .01**	5.9 ± 3.0 (7.8–13.4)	** < .01**	** < .01**
Left	5.6 ± 2.8 (0.3–13.7)		8.0 ± 3.0 (7.8–13.4)		** < .01**
Side to side Difference	1.5 ± 2.4 (-4.3–9.2)		2.0 ± 2.7 (-5.4–9.2)		NS

Values in bold indicate stastical significance (*P* < .05)

*PTS* posterior tibial slope, *aPTS* anterior PTS, *mPTS* middle PTS, *pPTS* posterior PTS;, *ACL* anterior cruciate ligament, *R* right, *L* left, *C* control, *A* ACL, *NS* Not Significant

^a^Data are expressed as mean ± SD (range)

**Table 4:** PTS comparison between injury and non-injury, dominant and non-dominant leg in ACL group^a^

	aPTS	*p*-value	mPTS	*p*-value	pPTS	*p*-value
Injury	12.0 ± 3.2 (5.4–23.4)	** < .01**	9.0 ± 2.9 (1–18)	** < .01**	7.5 ± 3.2 (0.1–17.8)	** < .01**
Non-Injury	11.1 ± 3.0 (2.3–18.6)		8.0 ± 2.9 (0.1–17.2)		6.4 ± 3.2 (-4.2 -13.8)	
Dominant	10.8 ± 3.3 (2.3–23.4)	** < .01**	7.7 ± 2.8 (0.1–17.8)	** < .01**	6.1 ± 3.2 (-4.2 -17.8)	** < .01**
Non-Dominant	12.3 ± 3.1 (4.2–20.8)		9.3 ± 2.8 (1.9 -18)		7.7 ± 3.1 (0.2–14.6)	

Values in bold indicate stastical significance (*P* < .05)

*PTS* posterior tibial slope, *aPTS* anterior PTS, *mPTS* middle PTS, *pPTS* posterior PTS, *ACL* anterior cruciate ligament, *NS* Not Significant

^a^Data are expressed as mean ± SD (range)

**Table 5:** PTS comparison between Male and Female in ACL group^a^

	aPTS	*p*-value	mPTS	*p*-value	pPTS	*p*-value
**Right**						
Male	11.0 ± 3.4	NS	7.7 ± 2.9	NS	6.2 ± 3.4	NS
Female	10.4 ± 2.8		7.4 ± 2.4		5.7 ± 2.7	
**Left**						
Male	13.2 ± 3.2	**0.02**	9.9 ± 2.7	NS	8.4 ± 3.2	NS
Female	12.3 ± 2.8		9.3 ± 2.6		7.7 ± 2.9	
**Injury**						
Male	12.4 ± 3.4	NS	9.2 ± 3.1	NS	7.8 ± 3.5	NS
Female	11.9 ± 2.9		8.9 ± 2.5		7.3 ± 2.9	
**Non-Injury**						
Male	11.7 ± 3.5	**0.02**	8.4 ± 2.9	NS	6.8 ± 3.4	**0.04**
Female	10.9 ± 2.9		7.9 ± 2.7		6.2 ± 2.9	
**Dominant**						
Male	11.3 ± 3.4	NS	8.0 ± 2.9	NS	6.5 ± 3.4	NS
Female	10.6 ± 3.0		7.6 ± 2.6		5.9 ± 2.8	
**Non-Dominant**						
Male	12.8 ± 3.4	NS	9.6 ± 2.9	NS	7.6 ± 2.8	NS
Female	12.2 ± 2.7		9.2 ± 2.5		8.1 ± 3.4	

Values in bold indicate stastical significance (*P* < .05)

*PTS* posterior tibial slope, *aPTS* anterior PTS, *mPTS* middle PTS, *pPTS* posterior PTS, *ACL* anterior cruciate ligament, *NS* Not Significant

^a^Data are expressed as mean ± SD (range)

There were 38 (20.3%) patients in the control group and 65 (34.7%) in the ACL injury group that demonstrated a difference in PTS of ≥ 3° (Table 6). There were significantly more patients with a greater PTS on the left than on the right among the control group (97.3% vs 2.6%; *p* < 0.01) and the ACL group (95.3% vs 4.7%; *p* < 0.01). Similar results were observed for the injured vs non-injured side (73.8% vs 26.2%; *p* < 0.01) and the non-dominant vs dominant side (86.1% vs 13.9%; *p* < 0.01) in the ACL injury group. The trend was similar for both males and females.

**Table 6:** Incidence of large mPTS difference^a^

	Total	*p*-value	Male	*p*-value	Female	*p*-value
**Control** (*n* = 38)						
Right ≧ Left (*n* = 1)	2.6%	** < .01**	0%	** < .01**	3.5%	** < .01**
Right ≦ Left (*n* = 37)	97.3%		100%		96.4%	
**ACL injury** (*n* = 65)						
Right ≧ Left (*n* = 3)	4.7%	** < .01**	4.1%	** < .01**	4.8%	** < .01**
Right ≦ Left (*n* = 62)	95.3%		95.8%		95.1%	
Injury ≦ Non-Injury (*n* = 17)	26.2%	** < .01**	29.1%	** < .01**	24.3%	** < .01**
Injury ≧ Non-Injury (*n* = 48)	73.8%		70.8%		75.6%	
Dominant ≧ Non-dominant (*n* = 9)	13.9%	** < .01**	16.6%	** < .01**	12.1%	** < .01**
Dominant ≦ Non-dominant (*n* = 56)	86.1%		83.3%		87.8%	

Values in bold indicate stastical significance (*P* < .05)

*PTS* posterior tibial slope, *mPTS* middle PTS, *ACL* anterior cruciate ligament

^a^Value are presented as percentage

Univariate linear regression analysis showed that the injured, non-dominant, and left sides were significantly associated with a larger mPTS (Table 7). After univariate linear regression analysis, variables such as gender, weight, injured side, non-dominant side, and left side were added to the multiple regression analysis as independent variables. Multiple regression analysis showed that only the left side was significantly associated with mPTS (*p* < 0.001).

**Table 7:** Uni & multiple logistic regression analysis for mPTS in ACL group^a^

Variable	Univariate			Multivariate		
	Odds ratio	95% CI	*p*-value	Odds ratio	95% CI	*p*-value
Age	-0.002	-0.022—-0.018	NS			
Sex, male	0.391	-0.218—1.001	NS	0.12	-0.542—0.786	NS
Body Height	0.018	-0.017- 0.055	NS			
Body weight	0.027	-0.001—0.055	NS	0.024	-0.006—0.054	NS
Injury side	0.91	7.717—8.520	**0.01**	0.465	-0.088—1.019	NS
Dominant side	-1.6	-2.163—-1.060	** < .01**	-0.063	-0.974—0.848	NS
Left side	1.89	1.452—2.529	** < .01**	1.828	0.900 – 2.756	** < .01**

*mPTS* middle posterior tibial slope, *ACL* anterior cruciate ligament, *NS* Not Significant

^a^Values in bold indicate stastical significance (*P* < .05)

## Discussion

In this study, there were significant left–right side differences in both the control and ACL groups and intra-group differences between the injured and non-injured as well as the dominant and non-dominant leg for the ACL-injured group. A case with laterality was demonstrated in 20.3% of patients in the control and 34.7% of patients in the ACL group. The incidence was significantly higher on the left side, as well as on the injured and non-dominant sides in the ACL group. In the multiple regression analysis, the left side had the greatest influence on the size of mPTS. This is a previously unverified finding and is crucial when considering the etiology, postoperative results, and postoperative rehabilitation intervention for ACL injury.

There are numerous studies on the association between ACL injury and PTS. Systematic reviews have shown that patients with ACL injuries have a 1.9°-2.2° greater PTS than healthy individuals, using X-ray images [26]. MRI findings have also confirmed that patients with ACL injuries have a greater PTS both medially and laterally, with a particularly strong influence on the lateral side. In various biomechanical studies, this has been believed to be due to an increase in the shear force that pushes the tibia forward when a compressive load is applied to the knee joint, resulting in greater anterior tibial displacement (ATT) [8, 14] and an increased load on the ACL itself. Motion analysis also shows that a large lateral posterior tilt induces internal rotation of the tibia during running and landing. Recently, it has also been shown that a large PTS in ACL-injured knees increases the load on the meniscus, increasing medial and lateral posterior root injuries [13, 18] and ramp lesions [17]. Therefore, PTS is an important anatomic parameter for knee ligament reconstruction, which requires further investigation.

In this study, the PTS was significantly greater on the left side (2.0), as well as on the injured (0.9–1.1) and non-dominant (1.5–1.6) sides, based on several PTS evaluations. This value corresponds with the difference reported in previous studies comparing healthy controls with patients with ACL injuries [22]. Most reports on ACL injuries and laterality have been related to the dominant leg. It has been reported that female soccer players have a greater knee valgus angle in the non-dominant leg during the single-leg vertical jump landing compared to males [15]. In soccer players with ACL non-contact injuries, a trend for injuries to occur on the dominant (kicking) leg in males and on the non-dominant (supporting) leg in females was reported [5]. Additionally, in recreational skiers, most female skiers experienced left-sided injuries (68%) that occurred in the non-dominant leg (63.1%) [19]. Negrete et al. found no association between the side of injury and the dominant leg but noted that women tended to injure on the left side more than men [16]. In summary, there is a tendency for more injuries to be observed in the non-dominant leg and left side in females. When the results of our study are taken into consideration, it suggests that in addition to differences in neuromuscular patterns during various movements between the sexes, anatomical differences between the left and right knees may have influenced the final results.

The results of this study are important in several aspects. Firstly, the fact that left–right differences in PTS exist within the same individual, with greater PTS on the injured side, may provide strong evidence supporting an association between ACL injury and PTS. Secondly, the left–right difference in PTS may have important implications for how training and postoperative rehabilitation in a particular sport is conducted, especially for female athletes. If one side of the leg has a greater PTS, it may be important to focus on training that leg. In addition, the way rehabilitation programs are conducted, return-to-play criteria, and prophylactic orthotics may also need to take into account the left–right differences in PTS in sports medicine that place more stress on certain lower legs. Thirdly, it is also known that the degree of PTS has an impact on postoperative outcomes [1, 6]; therefore, it is important to recognize that the results may be influenced by the left–right and dominant leg proportions of the subjects in the various studies.

Our study had several limitations. Firstly, it may be possible that only the evaluation of the X-ray image of the lateral proximal tibia may not reflect the functional PTS. However, there is currently no reference standard for measuring PTS. Therefore, three different measurement methods were used to increase the validity of the results. Secondly, in our study, the left–right differences in medial and lateral PTS were not evaluated, and this is an issue for future validation. Thirdly, this study was conducted only for the same Asian ethnic group, and future verification is needed to determine whether the same results can be obtained for other ethnic groups with different lifestyles. Fourth, this study’s control group comprised outpatients without various information (such as body height, weight, and leg dominance) and were strictly considered abnormal. However, the cases with no problems in the detailed medical interview, follow-up observation, and imaging examination were also included in this study, so there should be no major problems with the results.

In this study, all measurements had significantly greater PTSs on the left side for controls and patients with ACL injuries as well as on the injured side and the non-dominant foot for patients with ACL injuries. The rate of difference of ≥ 3° was a similar trend for control and patients with ACL injuries. It may be necessary to consider left–right differences in PTS for preoperative prophylaxis and postoperative rehabilitation and outcomes.
